# Efficient Electrochemical Synthesis, Antimicrobial and Antiinflammatory Activity of 2–amino-5-substituted- 1,3,4-oxadiazole Derivatives

**DOI:** 10.4103/0250-474X.73917

**Published:** 2010

**Authors:** S. Kumar, D. P. Srivastava

**Affiliations:** Department of Chemistry, Iswar Saran Degree College, Allahabad - 211 004, India; 1 Department of Zoology, S M M Tawn P G College, Ballia-277 001, India

**Keywords:** Acetic acid, controlled potential electrolysis, electrochemical, electrooxidation, green chemistry, oxadiazole, platinum electrode

## Abstract

An efficient electrochemical method for the preparation of 2-amino-5-substituted-1,3,4-oxadiazoles (4a-k) at platinum anode through the electrooxidation of semicarbazone (3a-k) at controlled potential electrolysis has been reported in the present study. The electrolysis was carried out in the acetic acid solvent and lithium perchlorate was used as supporting electrolyte. The products were characterized by IR,^1^H-NMR,^13^C-NMR, mass spectra and elemental analysis. The synthesized compounds were screened for their in vitro growth inhibiting activity against different strains of bacteria viz., *Klebsilla penumoniae, Escherichia coli, Bassilus subtilis* and *Streptococcus aureus* and antifungal activity against *Aspergillus niger* and *Crysosporium pannical* and results have been compared with the standard antibacterial streptomycin and antifungal griseofulvin. Compounds exhibits significant antibacterial activity and antifungal activity. Compounds 4a and g exhibited equal while 4c, d, i and j slightly less antibacterial activity than standard streptomycin. Compounds 4a and g exhibited equal while 4b, c, d, f and i displayed slightly less antifungal activity than standard griseofulvins.

5-substituted-2-amino-1,3,4-oxadiazoles have been found to exhibit diverse activities such as antibacterial[1,2], antiHIV[1], antifungal[3–5], antitubercular[6], virucidal[7], antimalarial[8,9], insecticidal[10], herbicidal[11], analgesic[12], antiinflammatory[13], muscle relaxant[14], anticonvulsant[15], sedative, hypnotic[16], anticancer[17] and inhibition of lipid peroxidation[18].

Literature synthesis of oxadiazoles (4) includes bromine oxidation of semicarbazide derivative and the cyclodesulfurization of acylthiosemicarbazide derivatives in solution using I_2_/NaOH or 1,3-dicyclohexylcarbodimide (DCC)[19–21], as well as mercury(II) acetate (Hg(OAc)_2_) or yellow mercury(II) oxide (HgO)[22–25]. All these methods are usually carried out in different synthetic steps that require very dangerous reagents such as bromine or a full equivalent of Hg(II) and produce undesirable mercury byproducts, which must then be removed and properly disposed off after the reaction is completed. Not only the handling of these reagents is difficult but also very hazardous to environment. From the first step to the last stage of the reaction including extraction and purification of the products there from demands great precautions.

In the context of green chemistry, some 5-substituted-2-amino-1,3,4-oxadiazoles (4) have been synthesized by electrooxidative cyclization of semicarbazone (3) as a new general environmentally benign synthetic method. The development of ecofriendly synthetic methods are the need of the hour. In this respect, organic synthesis involving multi-component reactions under reagent-free conditions is a basic protocol because multistep conventional synthesis produces considerable amounts of environmentally unfavorable wastes, mainly due to a series of complex isolation procedures often involving expensive, toxic and hazardous solvents and reagents after each step. The application of electricity as a non conventional energy source for activation of reactions in suitable solvents has now gained popularity over the usual homogeneous and heterogeneous reactions, as it provides chemical processes with special attributes, such as enhanced reaction rates, better selectivity, higher yield of pure products and several eco-friendly advantages. These reactions do not require oxidizing reagents and can be performed at ordinary room temperature.

## MATERIALS AND METHODS

### General experimental procedure:

Column chromatography was carried out by using Merck silica gel 60. The purity of the synthesized compounds were ascertained by TLC on pre-coated silica gel plates in various solvent systems using iodine vapors and UV light as detecting agent. The melting points were recorded on an electrothermal apparatus and were uncorrected. Infra red spectra were recorded on a Shimadzu 8201 PC IR spectrophotometer in KBr pellets and reported in cm^-1^.^1^H NMR and^13^C NMR spectra were measured on Bruker DRX 300 MHz FT spectrometer instruments using DMSO-d_6_ as solvent with TMS and CDCl_3_ as internal standards (chemical shift in δ ppm). Carbon multiplities were assigned by DEPT techniques. The structures of the newly synthesized compounds were assigned on the basis of elemental analysis and were recorded on a Elementar Vario EL III. Carbon, hydrogen and nitrogen analyses were within ±0.4% of the theoretical values. Mass spectra were taken out on a Jeol SX 102/DA-6600 mass spectrometer using Argon/Xenon (6 KV, 10 mA) as the FAB gas. All the chemicals used were of synthetic and AR grade and was procured from Agros-Organics, USA, S. D. Fine Chem. Ltd., Mumbai and Merck, Mumbai, India.

### Synthesis of 2-amino-5-(*p*-bromophenyl)-1,3,4-oxadiazoles:

Semicarbazide hydrochloride (1.0 g, 8.96 mmol) (2) and NaOAc (12.2 mmol) was dissolved in (10 ml) water and then an aldehyde (0.5 g, 3.04 mmol) (1) was added with continuous stirring. The mixture was left overnight, which evolved a solid product semicarbazone (3) which was used as initial compound for the electrolysis. Semicarbazone (1.0 g, 4.52 mmol) (3) and LiClO_4_(0.106 g, 0.67 mmol) were dissolved in (100 ml) acetic acid.

### Electrolysis:

Preparative scale controlled potential electrolysis[26–30] were performed at room temperature in 250 ml three-electrode cell assembly with platinum plate as working as well as counter electrode and saturated calomel electrode as reference electrode. Magnetic stirrer was used for the proper mixing of reaction mixture. All the electrolysis experiments were carried out at their corresponding oxidation potentials and were completed in 3 to 5 h. After which no oxidation product was seen to diffuse in the bulk. All the products were solid and coloured and entirely different from the starting compound. The current potential data was recorded with the help of potentiostat at the interval of 15 min and has been depicted in Table 1. Approximately 4-6.5 Fmol^-1^of electricity was passed for the electrolysis which is very small in comparison to energy used in other conventional methods. The physical and analytical data of the synthesized compounds (4a-k) has been depicted in the Table 2. The spectral (IR,^1^H NMR,^13^C NMR and MS) and analytical data are in good agreement with their structures.

**TABLE 1:** ELECTROORGANIC SYNTHESIS OF 5-SUBSTITUTED-2-AMINO-1,3,4-OXADIAZOLES (4A-K)

Entry	R	Time (h)	Applied Potential (mV)	Current (mA)	Yield (%)
4a	*o*-BrC_6_H_4_	4	1540	110	88
4b	*m*-BrC_6_H_4_	5	2100	150	96
4c	*p*-BrC_6_H_4_	5	2250	120	86
4d	*o*-(NO_2_)C_6_H_4_	3	1850	90	92
4e	3-Pyridinyl	4	1800	70	79
4f	CH_2_Cl	5	2000	120	75
4g	CHCl_2_	5	1900	80	81
4h	*p*-(CH_3_)C_6_H_4_	3	1450	90	85
4i	3,4,5-(OCH_3_)3C_6_H_2_	5	1700	80	92
4j	1-C_10_H_7_	4	1600	100	87
4k	2-C_10_H_7_	4	2200	120	86

**TABLE 2:** PHYSICAL AND ANALYTICAL DATA OF 5-SUBSTITUTED-2-AMINO-1,3,4-OXADIAZOLE (4a-k)

Compound	R	Mol. for/ Mol. wt	Elemental analysis found (calcd) %			
			C	H	N	Br/Cl
4a	*o*-BrC_6_H_4_	C_8_H_6_N_3_OBr/240.01	39.52 (40.00)	2.40 (2.50)	17.35 (17.50	33.12 (33.33)
4b	*m*-BrC_6_H_4_	C_8_H_6_N_3_OBr/240.01	39.56 (40.00)	2.42 (2.50)	17.35 (17.50)	33.22 (33.33)
4c	*p*-BrC_6_H_4_	C_8_H_6_N_3_OBr/240.01	39.53 (40.00)	2.41 (2.50)	17.35 (17.50)	33.15 (33.33)
4d	*o*-NO_2_C_6_H_4_	C_8_H_6_N_5_O_5_ /252.12	37.89 (38.09)	2.40 (2.38)	27.35 (27.77)	-
4e	3-Pyridinyl	C_7_H_6_N_4_O/162.15	51.35 (51.85)	3.40 (3.70)	34.58 (34.57)	-
4f	CH_2_Cl	C_3_H_4_N_3_OCl/133.53	26.56 (26.96)	2.59 (2.69)	31.15 (31.46)	26.60 (26.59)
4g	CHCl_2_	C_3_H_3_N_3_OCl_2_/167.9	21.35 (21.55)	1.68 (1.70)	25.14 (25.14)	41.66 (41.91)
4h	*p*-(CH_3_)C_6_H_4_	C_9_H_9_N_3_O/175.19	61.52 (61.71)	5.11 (5.14)	23.85 (24.00)	-
4i	3,4,5-(OCH_3_)3C_6_H_2_	C_11_H_13_N_3_O_4_/251.24	52.40 (52.59)	5.11 (5.17)	16.52 (16.73)	-
4j	1-C_10_H_7_	C_12_H_9_N_3_O_2_/227.22	67.92 (68.24)	4.26 (4.26)	19.85 (19.90)	-
4k	2-C_10_H_7_	C_12_H_9_N_3_O_2_/227.23	67.95 (68.24)	4.25 (4.26)	19.86 (19.90)	-

### 2-amino-5-(*o*-bromophenyl)-1,3,4-oxadiazole (4a):

Brownish crystals; mp: 68-69°; IR (KBr, cm^-1^): 3360 (NH), 3045 (C-H aromatic), 1613 (C=N-N=C), 1470 (C=C aromatic), 1265, 1072 (C-O-C), 980, 890, 750, 595 (substituted benzene);^1^H NMR (DMSO-d_6_, δ ppm): 7.75 (s, 2H, NH_2_), 6.94-7.14 (m, 4H, aromatic H);^13^C NMR (DMSO-d_6_, δ ppm): 158.9 (C), 147.7 (C), 140.6 (C), 132.9 (CH), 131.9 (CH), 125.1 (C), 115.4 (C), 106.4 (CH); MS m/z: 240 (M^+^), 241 (M^+^+ 1, 100%) for C_8_H_6_N_3_OBr.

### 2-amino-5-(*m*-bromophenyl)-1,3,4-oxadiazole (4b):

Brownish crystals; mp: 75-76°; IR (KBr, cm^-1^): 3362 (NH), 3042 (C-H aromatic), 1620 (C=N-N=C), 1473 (C=C aromatic), 1265, 1072 (C-O-C), 980, 890, 7505, 595 (substituted benzene);^1^H NMR (DMSO-d_6_, δ ppm): 7.75 (s, 2H, NH_2_), 6.94-7.24 (m, 4H, aromatic H);^13^C NMR (DMSO-d_6_, δ ppm): 158.9 (C), 147.7 (C), 140.6 (C), 132.9 (CH), 131.9 (CH), 125.1 (C), 115.4 (C), 106.4 (CH); MS m/z: 240 (M^+^), 241 (M^+^+ 1, 100%) for C_8_H_6_N_3_OBr.

### 2-amino-5-(*p*-bromophenyl)-1,3,4-oxadiazole (4c):

Brownish crystals; mp: 69-70°; IR (KBr, cm^-1^): 3360 (NH), 3045 (C-H aromatic), 1615 (C=N-N=C), 1475 (C=C aromatic), 1275, 1075 (C-O-C), 985, 890, 755, 597 (substituted benzene);^1^H NMR (DMSO-d_6_, δ ppm): 7.75 (s, 2H, NH_2_), 7.71 (d, 2H, aromatic H), 7.64 (d, 2H, aromatic H);^13^C NMR (DMSO-d_6_, δ ppm): 158.9 (C), 147.7 (C), 140.6 (C), 132.9 (CH), 131.9 (CH), 125.1 (C), 115.4 (C), 106.4 (CH); MS m/z: 240 (M^+^), 241 (M^+^+ 1, 100%) for C_8_H_6_N_3_OBr.

### 2-amino-5-(*o*-nitrophenyl)-1,3,4-oxadiazole (4d):

Dark yellowish needles; mp: 71-73°; IR (KBr, cm^-1^): 3341 (NH), 3035 (C-H aromatic), 1607 (C=N-N=C), 1550 (N=O), 1465 (C=C aromatic),1275, 1070 (C-O-C), 985, 865, 810, 730 (substituted benzene);^1^H NMR (DMSO-d_6_, δ ppm): 7.75 (s, 2H, NH_2_), 7.25-7.69 (m, 4H, aromatic H);^13^C NMR (DMSO-d_6_, δ ppm): 171.3 (C), 147.1 (C), 146.8 (C), 140.8 (C), 136.8 (C), 134.7 (CH), 130.4 (CH), 121.2 (CH); MS m/z: 252 (M^+^), 253 (M^+^+ 1, 100%) for C_8_H_6_N_5_O_5_.

### 2-amino-5-(3-pyridinyl)-1,3,4-oxadiazole (4e):

Dark yellowish crystals; mp: 67-68°; IR (KBr, cm^-1^): 3350 (NH), 3037 (PyC-H), 1628 (C=N-N=C), 1472 (C=C aromatic), 1430-1600 (C=C and C=N str), 1070 (C-O-C);^1^H NMR (DMSO-d_6_, δ ppm): 7.18-8.56 (m, 4H, aromatic H), 7.75 (s, 2H, NH_2_);^13^C NMR (DMSO-d_6_, δ ppm): 171.2(C), 167 (C), 149.8 (CH), 135.7 (CH), 129 (C), 123.6 (CH), 122.1 (CH); MS m/z: 162 (M^+^), 163 (M^+^+ 1, 100%) for C_7_H_6_N_4_O.

### 2-amino-5-chloromethyl-1,3,4-oxadiazole (4f):

Brownish crystals; mp: 61-62°; IR (KBr, cm^-1^): 3360 (NH), 3062 (C-H), 1618 (C=N-N=C), 1280, 1066 (C-O-C), 680 (C-Cl);^1^H NMR (DMSO-d_6_, δ ppm): 7.75 (s, 2H, NH_2_), 3.8 (s, 2H, CH_2_);^13^C NMR (DMSO-d_6_, δ ppm): 170.6 (C), 167.6 (C), 24.9 (CH_2_); MS m/z: 133.5 (M^+^), 134.5 (M^+^+ 1, 100%) for C_3_H_4_N_3_OCl.

### 2-amino-5-dichloromethyl-1,3,4-oxadiazole (4g):

Brownish crystals; mp: 64-65°; IR (KBr, cm^-1^): 3360 (NH), 3065 (C-H), 1609 (C=N-N=C), 1280, 1066 (C-O-C), 690 (C-Cl);^1^H NMR (DMSO-d_6_, δ ppm): 7.75 (s, 2H, NH_2_), 3.9 (s, 1H, CH);^1^H NMR (DMSO-d_6_, δ ppm): 172.1 (C), 163.2 (C), 51.2 (CH); MS m/z: 167 (M^+^), 168 (M^+^+ 1, 100%) for C_3_H_3_N_3_OCl_2_.

### 2-amino-5-(*p*-methylphenyl)-1,3,4-oxadiazole (4h):

Light brown needles; mp: 74-75°; IR (KBr, cm^-1^): 3270 (NH), 3045 (C-H aromatic), 3010 (C-H), 2927, 1602 (C=N-N=C), 1473 (C=C aromatic), 1265, 1069 (C-O-C), 960, 765 (substituted benzene); H^1^NMR (400 MHz, CDCl_3_) 7.75 (s, 2H, NH_2_), 7.69 (d, 2H, aromatic H), 7.62 (d, 2H, aromatic H), 1.12 (s, 3H, CH_3_); C^13^NMR (75 MHz, CDCl_3_) 178.2(C), 149.9 (C), 141.3(CH), 138.6 (CH), 136.5 (CH), 129 (CH), 127.8 (CH), 126.5 (CH), 20.6 (CH_3_); MS m/z: 175 (M^+^), 176 (M^+^+ 1, 100%) for C_9_H_9_N_3_O.

### 2-amino-5-(3,4,5-trimethoxyphenyl)-1,3,4-oxadiazole (4i):

Dark brownish needles; mp: 84-85°; IR (KBr, cm^-1^): 3261 (NH), 3045 (C-H aromatic), 2815 (OCH_3_), 1609 (C=N-N=C), 1470 (C=C aromatic), 1270, 1069 (C-O-C), 915, 870, 790 (substituted benzene);^1^H NMR (DMSO-d_6_, δ ppm): 7.75 (s, 2H, NH_2_), 6.46-7.70 (m, 2H, aromatic H), 3.11 (s, 9H, OCH_3_);^13^C NMR (DMSO-d_6_, δ ppm): 172.4 (C), 167.5 (C), 146.7 (C), 146.3 (C), 134.9 (C), 129.5 (C), 105.7 (CH), 106.5 (CH), 54.3 and 44.6 (CH_3_); MS m/z: 251 (M^+^), 252 (M^+^+ 1, 100%) for C_11_H_13_N_3_O_4_.

### 2-amino-5-(1-naphthyl)-1,3,4-oxadiazole (4j):

Dark brownish needles; mp: 94-95°; IR (KBr, cm^-1^): 3330 (NH), 3045 (C-H aromatic), 1612 (C=N-N=C), 1475 (C=C aromatic), 1055 (C-O-C), 775 (substituted aromatics);^1^H NMR (DMSO-d_6_, δ ppm): 7.75 (s, 2H, NH_2_), 7.25-7.69 (m, 7H, aromatic H);^13^C NMR (DMSO-d_6_, δ ppm): 171.5 (C), 149.5 (C), 133.7 (C), 128 (CH), 126 (C); MS m/z: 227 (M^+^), 228 (M^+^+ 1, 100%) for C_12_H_9_N_3_O_2_.

### 2-amino-5-(2-naphthyl)-1,3,4-oxadiazole (4k):

Dark brownish needles; mp: 96-97°; IR (KBr, cm^-1^): 3335 (NH), 3045 (C-H aromatic), 1622 (C=N-N=C), 1468 (C=C aromatic), 1045 (C-O-C), 775 (substituted aromatics);^1^H NMR (DMSO-d_6_, δ ppm): 7.75 (s, 2H, NH_2_), 7.25-7.69 (m, 7H, aromatic H);^13^C NMR (DMSO-d_6_, δ ppm): 171.6 (C), 149.5 (C), 133.7 (C), 128 (CH), 126 (C); MS m/z: 227 (M^+^), 228 (M^+^+ 1, 100%) for C_12_H_9_N_3_O_2_.

### Screening for Antimicrobial activity:

All the synthesized compounds were tested for antimicrobial activity by adopting the experimental method of Benson[31]. Whatman No.1 filter paper discs of 6 mm diameter, placed in a Petri dish, were autoclaved. The test compounds in measured quantities (1.0 mg, 0.5 mg) were dissolved in 5 ml dimethylformamide to produce 200 ppm and 100 ppm solutions, respectively. The filter paper discs were allowed to dry and the amount of the substance per disc was taken as 500 and 250 µg. The bacterial (24 h) and fungal (48 h) cultures from the slants were diluted with sterile water and mixed thoroughly to prepare a clear homogeneous suspension. These suspensions were uniformly spread on solidified agar (nutrient and potato dextrose agar) medium. The filter paper discs prepared from dimethylformamide medium were carefully placed over the spread cultures and incubated at 37° for 24 h for bacteria and at 28-30° for 48 h for fungi. Paper discs treated with dimethylformamide alone served as control. After the incubation period the plates were examined for inhibition zones. The diameters of inhibition zones (including the diameter of the disc) were measured. All determinations were made in triplicate for each of the compounds and the average value was taken. The antibacterial and antifungal screening results were presented in Tables 3 and 4.

**TABLE 3:** ANTIBACTERIAL SCREENING RESULTS OF COMPOUNDS (4a-k)

Compound	Zone of inhibition (mm)							
	*E. coli* (ATCC 25922)		*K. pneumonia* (CIP 53153)		*B. subtilis* (ATCC 6633)		*S. aureus* (ATCC 25923)	
	25 ppm	50 ppm	25 ppm	50 ppm	25 ppm	50 ppm	25 ppm	50 ppm
4a	18	21	17	23	18	24	17	22
4b	4	6	4	7	5	7	4	6
4c	15	18	3	20	14	19	13	19
4d	12	17	9	13	12	15	11	15
4f	16	20	17	21	15	20	14	19
4g	17	21	14	20	16	20	16	20
4i	15	18	14	18	13	17	17	19
Streptomycin	20	23	19	24	19	24	19	23

**TABLE 4:** ANTIFUNGAL SCREENING RESULTS OF COMPOUNDS (4a-k)*

Compound	*A. niger* (ATCC 16404)			*C. pannical* (ATCC 10231)		
	10 ppm	100 ppm	1000 ppm	10 ppm	100 ppm	1000 ppm
4a	18	43	76	19	43	78
4b	15	38	65	16	36	67
4c	44	58	98	45	57	98
4d	21	46	75	24	43	78
4f	38	56	97	40	51	96
4g	40	53	96	42	53	97
4i	21	44	70	20	43	68
Griseofulvin	66	86	100	65	83	100

* Average inhibition of fungal growth (%) at stated concentration (mg/liter-1)

### Antiinflammatory activity against carrageenan-induced rat paws oedema:

Antiinflammatory activity was determined by carrageenan-induced rat paw method of Winter[32]. Male Wistar rats (125-150 g) were used for the experiment. They were fed with standard pellet diet and water was given *ad libitum*. The animals were acclimatized for one week under laboratory conditions before performing the test. They were housed in polypropylene cages under standard conditions (30±1°, 12/12 h light/dark cycles on 60-70% RH). The standard groups received phenylbutazone 50 mg/kg body weight po, suspended in 1% w/v of carboxymethylcellulose (CMC) in distilled water. The test group received synthesized compounds (4a-k) (50 mg/kg body weight po, suspended in 1% w/v of CMC in water). The control group received corresponding amount of vehicle (1% w/v of CMC). All the test compounds and standard drug were administered 30 min prior to carrageenan injection.

Acute edema was induced in the right hind paw of rats by injecting 0.1 ml of freshly prepared 1% w/v of aqueous solution of carrageenan (Sigma, USA) in the subplanter region of right hind paw. After the carrageenan injection the paw volume was measured before and after 1, 2 and 3 h by plethysmometer (UGO-Basile, Italy). The difference between the left and right paw was taken as a measure of oedema. Any significant reduction in the volume of the paw compared to the control group was considered as anti-inflammatory response. Percent inhibition of inflammation after 3 h was calculated by applying Newbould formula. % inhibition = 100×1–(a – x)/(b – y), where, x= mean paw volume of rats before the administration of carrageenan injection in the test and the standard groups, y = mean paw volume of rats before the administration of carrageenan injection in the control group, a = mean paw volume of rats after the administration of carrageenan and test compound or standard compound, b = mean paw volume of rats after the administration of carrageenan injection in control group. The results are presented in Table 5.

**TABLE 5:** ANTIINFLAMMATORY ACTIVITY OF COMPOUNDS (4a-k)

Compounds	Normal paw volume (x)	Paw oedema 3h after carrageenan injected (a)	% inhibition of oedema (1-(a-x)/(b-y))×100
4a	0.66±0.04	0.85±0.03	46.41
4b	0.70±0.03	0.91±0.03	24.12
4c	0.68±0.03	0.88±0.04	35.62
4d	0.69±0.04	0.89±0.03	32.38
4e	0.72±0.03	0.93±0.03	21.12
4f	0.66±0.04	0.91±0.04	35.78
4g	0.65±0.03	0.90±0.03	42.21
4h	0.67±0.02	0.87±0.02	44.12
4i	0.715±0.05	0.945±0.04	20.78
4j	0.70±0.04	0.93±0.05	25.00
4k	0.71±0.03	0.92±0.05	24.85
Control	0.68±0.03 (y)	0.98±0.04 (b)	-
Phenylbutazone	0.68± 0.04	0.84±0.03	54.12

^*^P<0.01, vs standard mean±SEM

## RESULTS AND DISCUSSION

**Scheme 1:**
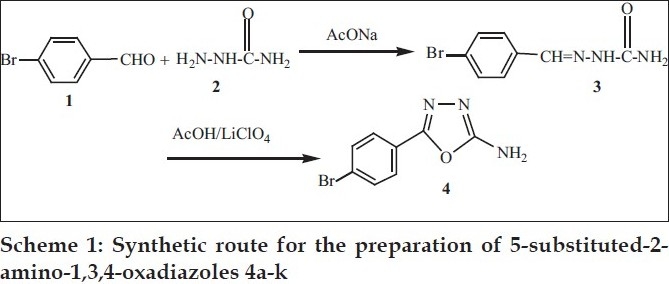
Synthetic route for the preparation of 5-substituted-2-amino-1,3,4-oxadiazoles 4a-k

It is obvious that an environmentally benign synthetic method for the synthesis of 2-amino-5-substituted-1,3,4-oxadiazoles would be highly attractive. Keeping this objective in the mind, we have synthesized 2-amino-5-substituted-1,3,4-oxadiazole 4 derivatives through the electrooxidation of semicarbazone 3 at a platinum electrode in controlled potential electrolysis in acetic acid (Scheme 1). This electrochemical cyclization evolves the oxadiazoles without requirement of any hazardous reagents. We have used acetic acid as a solvent and lithium perchlorate (LiClO_4_) as an electrolyte that can be handled very easily without major precautions.

**Scheme 2:**
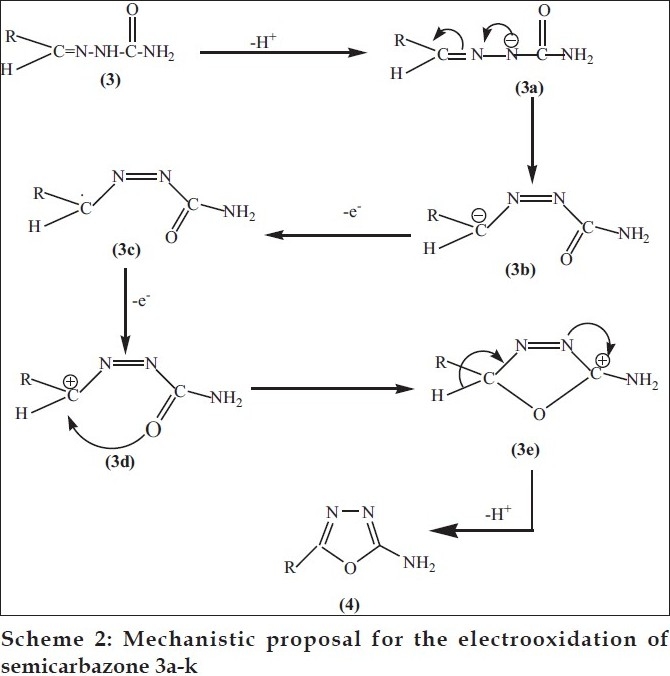
Mechanistic proposal for the electrooxidation of semicarbazone 3a-k

The first step of the mechanism (Scheme 2) represents the deprotonation of 3 to form an anion 3a, which rearranges itself into 3b and evolves a free radical 3c after one electron oxidation. Subsequent second electron oxidation from the free radical 3c gives a carbocation 3d. The formation of carbon oxygen bond completes the ring. On loosing a proton in the last step 2-amino-5-substituted-1,3,4-oxadiazole 4 was obtained.

The antimicrobial activity indicated that compounds 4a, b, c, d, f, g and i were found to be active against *Klebsilla penumoniae, Escherichia coli, Basillus subtilis, Streptococus aureus* organisms at the two concentrations (25 and 50 ppm) taking Streptomycin as the standard. The majority of the compounds exhibited significant antibacterial activity against *E. coli, K. pneumonia, B. subtilis* and *S. aureus* as compared to standard streptomycin. The screening results of antibacterial activity revealed that compound 4a and g exhibited approximately similar activity to the standard Streptomycin. Compounds 4c, d, i and j exhibited slightly less antibacterial activity. Compound 4b exhibited weak while other compounds have zero or negligible antibacterial activity against all bacterial strains used for our evaluation. The screening results showed that compounds 4b, c d, f and i displayed better antifungal activity against *Aspergillus niger* and *C. pannical* along with the standard fungicide Griseofulvins. The screening results revealed that compounds 4a and g showed equal antifungal activity when compared with the griseofulvins.

The antimicrobial activity of the compounds varied upon the type and position of the substituents at 5-substituted-2-amino-1,3,4-oxadiazole moiety. It can be concluded from the antimicrobial screening results that when 5-substituted-2-amino-1,3,4-oxadiazoles were substituted with aryl halide the antimicrobial activity was altered to an appreciable extent.

The results of antiinflammatory activity (Table 5) shows that the compounds 4d and 4h were active (p<0.01) with the standard. Moreover, compounds 4c, d, f and g showed significant antiinflammatory activity (p<0.01). Carrageenan-induced paw edema was taken as a prototype of exudative phase of inflammation. The development of edema has been described as biphasic. The initial phase was due to the release of histamine, serotonins, 5-hydroxy tryptamine and kinins in the first hour after injection of carrageenan. The second phase was related to the release of prostaglandin[33–35] like substances in 2-3 h. Hence, the significant anti-inflammatory effect may be due to an inhibitory effect exerted predominantly on the mediators of inflammation induced by phlogogenic stimuli.
